# Partially Glycosylated Dendrimers Block MD-2 and Prevent TLR4-MD-2-LPS Complex Mediated Cytokine Responses

**DOI:** 10.1371/journal.pcbi.1002095

**Published:** 2011-06-30

**Authors:** Teresa S. Barata, Ian Teo, Steve Brocchini, Mire Zloh, Sunil Shaunak

**Affiliations:** 1 Center for Structural Chemistry, School of Pharmacy, University of London, London, United Kingdom; 2 Departments of Medicine, Infection & Immunity, Imperial College London, London, United Kingdom; Centro de Investigación Príncipe Felipe (CIPF), Spain

## Abstract

The crystal structure of the TLR4-MD-2-LPS complex responsible for triggering powerful pro-inflammatory cytokine responses has recently become available. Central to cell surface complex formation is binding of lipopolysaccharide (LPS) to soluble MD-2. We have previously shown, in biologically based experiments, that a generation 3.5 PAMAM dendrimer with 64 peripheral carboxylic acid groups acts as an antagonist of pro-inflammatory cytokine production after surface modification with 8 glucosamine molecules. We have also shown using molecular modelling approaches that this partially glycosylated dendrimer has the flexibility, cluster density, surface electrostatic charge, and hydrophilicity to make it a therapeutically useful antagonist of complex formation. These studies enabled the computational study of the interactions of the unmodified dendrimer, glucosamine, and of the partially glycosylated dendrimer with TLR4 and MD-2 using molecular docking and molecular dynamics techniques. They demonstrate that dendrimer glucosamine forms co-operative electrostatic interactions with residues lining the entrance to MD-2's hydrophobic pocket. Crucially, dendrimer glucosamine interferes with the electrostatic binding of: (i) the 4′phosphate on the di-glucosamine of LPS to Ser118 on MD-2; (ii) LPS to Lys91 on MD-2; (iii) the subsequent binding of TLR4 to Tyr102 on MD-2. This is followed by additional co-operative interactions between several of the dendrimer glucosamine's carboxylic acid branches and MD-2. Collectively, these interactions block the entry of the lipid chains of LPS into MD-2's hydrophobic pocket, and also prevent TLR4-MD-2-LPS complex formation. Our studies have therefore defined the first nonlipid-based synthetic MD-2 antagonist using both animal model-based studies of pro-inflammatory cytokine responses and molecular modelling studies of a whole dendrimer with its target protein. Using this approach, it should now be possible to computationally design additional macromolecular dendrimer based antagonists for other Toll Like Receptors. They could be useful for treating a spectrum of infectious, inflammatory and malignant diseases.

## Author Summary

Dendrimers are well-defined branched symmetrical macromolecules. In biologically based experiments, we have shown that a generation 3.5 PAMAM dendrimer whose surface was modified with 8 surface glucosamine molecules inhibited TLR4 mediated cytokine inflammation in both primary human cells and a clinically validated rabbit model of tissue scaring. Molecular dynamics simulations also showed that these molecules had the flexibility, surface electrostatic charge, and hydrophilicity to make them therapeutically useful antagonists. Central to the TLR4-MD-2-LPS cell surface complex is binding of lipopolysaccharide (LPS) to soluble MD-2. We now show that dendrimer glucosamine forms co-operative electrostatic interactions with residues lining the entrance to MD-2's hydrophobic pocket. These interactions block the entry of the lipid chains of LPS into MD-2's hydrophobic pocket and prevent complex formation. We have therefore defined the first nonlipid-based synthetic MD-2 antagonist using both animal model based studies and molecular modelling studies of a whole dendrimer with its target protein. Using this approach, it should now be possible to computationally design additional macromolecular dendrimer based antagonists for other Toll Like Receptors. They could be useful for treating a spectrum of infectious, inflammatory and malignant diseases.

## Introduction

Dendrimers are a class of spherical macromolecules that can be distinguished from conventional linear polymers by their highly branched and symmetrical architecture. Polyamidoamine (PAMAM) dendrimers are, by far, the best studied of the commercialised and divergently synthesised dendrimers. Typically, these dendrimers are available in whole generations (amine terminated) and half-generations (carboxylic acid terminated) that are representative of both their size (i.e., diameter in angstroms) and molecular weight [1]–[3]. They can be made by controlled sequential processes to give well defined chemical structures. Even at low concentrations, the peripheral amine groups of cationic dendrimers damage cell membranes and lead to cell toxicity [4]. In contrast, anionic dendrimers have:- (i) physico-chemical properties that are similar to those of conventional small molecule drugs; (ii) can be modified to exist as zwitterions at physiological pH; (iii) have considerable buffering capacity that makes them physico-chemically “similar” to albumin, and therefore biocompatible. However, unlike proteins, they:- (a) do not undergo proteolytic degradation in plasma; (b) are not immunogenic or otherwise toxic even after repeated administration by various routes to animals; (c) can be optimized for their circulation time; (d) show preferential accumulation in tissues containing inflammatory cells compared to healthy tissue at a ratio of 50∶1 [4]. In addition, the National Cancer Institute's Nanotechnology Characterisation Laboratory recently undertook detailed chemical and toxicological characterization of anionic PAMAM dendrimers and found them to be both stable and biocompatible [5]. Taken together, these observations suggest that anionic dendrimer based drugs could become a new and safe class of “synthetic baby-bio” (SBB) drugs.

In biologically based experiments, we have already shown that a generation (G) 3.5 PAMAM dendrimer that was partially modified with an average of 8 surface glucosamine molecules inhibited TLR4-MD-2-LPS pro-inflammatory cytokine mediated inflammation in primary human monocytes, dendritic cells, and in a clinically validated rabbit model of tissue scaring [6]. Molecular modeling studies suggested, and experimental studies confirmed, that the surface loading of a G3.5 PAMAM dendrimer (with an average of 64 peripheral carboxylic acid groups) could not be increased beyond an absolute maximum cluster density of 12 evenly spaced surface glucosamine molecules using the divergent synthesis approach [7]. Frontier molecular orbital theory (FMOT) and molecular dynamics simulations also showed that the optimum surface loading and distribution of the zero length amide bond conjugated glucosamine molecules was determined by both electronic effects and the different dynamic conformations adopted by the modified dendrimer during the incremental addition of glucosamine [7], [8]. Importantly, the structural features and the dynamic behavior of this partially glycosylated dendrimer showed that its flexibility and polarity changed with the incremental addition of glucosamine molecules. Notably, these surface glucosamine molecules remained available for interaction with the biological target.

Innate immunity provides immediate and efficient defence against microbial infection and tissue injury by promoting pro-inflammatory cytokine responses that induce adaptive immune responses. Toll-like receptors (TLRs), a family of type I transmembrane glycoproteins, are central to these vertebrate innate immune responses because they recognize a broad range of soluble microbial stimuli on pathogens [9]. They are therefore called pattern recognition receptors. Their extracellular segments consist of leucine-rich repeats (LRR) with horseshoe-like shapes [10]. The binding of soluble agonist ligands leads to protein conformational change, receptor complex rearrangement, recruitment of specific adaptor proteins to the intracellular domain, and the initiation of signalling cascades.

Lipopolysaccharide (LPS – variable MWt>10 kDa) is the outer membrane glycolipid of Gram-negative bacteria that induces this innate immune response [11]. It is composed of:- (a) the hydrophilic polysaccharide core; (b) the solvent exposed hydrophobic lipid A component; (c) the O-antigen. Only the lipid A is required to induce pro-inflammatory cytokine responses. It is composed of a diphosphorylated β-1,6-linked D-glucosamine disaccharide linked via amide or ester bonds to 3-hydroxy fatty acids further substituted by nonhydroxylated 12–14 carbon fatty acid chains [12]. The cell surface interaction between LPS, TLR4 and MD-2 protein is central to the initiation of pro-inflammatory cytokine mediated responses. The transport protein CD14 first collects and delivers soluble LPS to soluble, circulating and monomeric MD-2 [13], [14]. The two phosphorylated glucosamine residues of lipid A (MWt∼2 kDa) bind via electrostatic interactions to the charged entrance (Ser118 {for the 4′ phosphate} and Lys122 {for the 1′ phosphate}, and to Arg90 and Lys91) of human MD-2's hydrophobic pocket. The diglucosamine and phosphate groups remain in solution and outside the hydrophobic pocket; these residues serve to anchor LPS to the entrance of MD-2's hydrophobic pocket. This is followed by the lipid chains of LPS becoming buried in MD-2's hydrophobic pocket [15]. Subsequent formation of the human TLR4-MD-2-LPS complex requires:- (a) hydrophobic interactions between Met85, Leu87, Ile124 and Phe126 on human MD-2 with Phe436, Phe436, Phe440 and Phe444 respectively on human TLR4; (b) hydrogen bond interactions between Arg90 and Gly123 on human MD-2 with Glu439 and Ser416 respectively on TLR4 [16]. The complex formed undergoes conformational changes that lead to receptor complex dimerization, triggering of intracellular signaling events, and the initiation of pro-inflammatory cytokine production [17], [18]. Low level stimulation of pro-inflammatory cytokine production is physiologically beneficial for dealing with infections because it enables the activation of co-stimulatory molecules and the generation of adaptive immune responses. However, excess pro-inflammatory cytokine production can become pathological with serious adverse effects on the host which include septic shock and death [19].

Previous glycodendrimer based studies have used fully glycosylated molecules; i.e., all of the peripheral groups of the dendrimer linked to saccharides using a spacer arm derived amide bond [20], [21]. In contrast, our studies are the first to use a partially glycosylated dendrimer; i.e., a small number of glucosamine molecules linked to the dendrimer's surface using a zero length amide bond. In order to better understand the molecular mechanism responsible for the ability of partially glycosylated dendrimers to block pro-inflammatory cytokine production, we studied their interaction with the LPS recognition system using molecular modelling techniques. We started from the premise that their inherent flexibility and, potentially, their size (MWt = 13.6 kDa for a G3.5 PAMAM dendrimer) were important molecular determinants of their biological properties [6]. However, the major limitation for structural biology studies was the absence of structural data. Although molecular dynamics simulation based study of dendrimer-biomacromolecules interactions is possible [22], [23], we did not have any information about the initial complex or a starting point for the simulation. We therefore used molecular docking as a tool to investigate the interactions between the partially glycosylated dendrimer and the LPS recognition system. To our knowledge, no previous molecular modelling study has reported the docking of a whole dendrimer with a protein target. Our studies therefore provide new insights that should enable the design of new macromolecules as novel antagonists of biologically important Toll like Receptor-ligand interactions [24].

## Results/Discussion

We first chose and validated molecular docking software packages that used protein as the target and synthetic macromolecules as the ligand. These are described in the Methods section and results shown in the Supplementary Figures S1, S2, S3, S4, S5, S6, and S7. Amongst docking softwares that deal with ligands of increasingly larger size, we found that Patchdock [25], Hex [26] and Glue [27] could be used. These packages were validated by reproducing the correct orientations of the ligands in:- (i) the crystal structure of the mouse TLR4-MD-2 complex (PDB entry: 2z64); (ii) the crystal structure of lipid A complexed with human MD-2 (PDB entry: 2z59).

**Figure 1:**
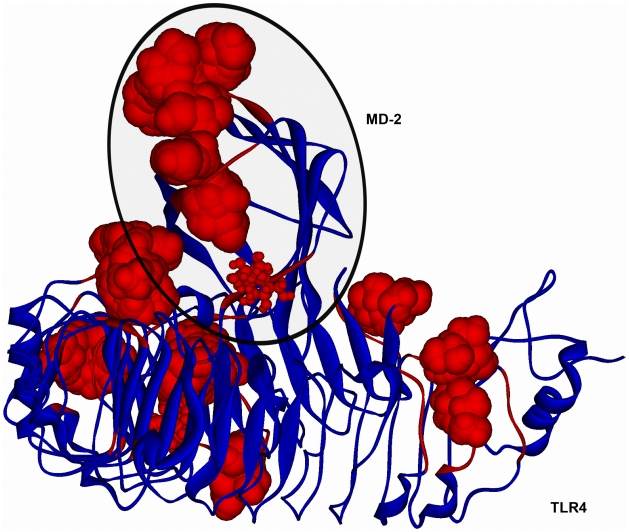
Solutions of the docking studies between the human TLR4-MD-2 complex and glucosamine molecules performed with GRID protocol. The human TLR4-MD-2 complex is shown with its points of interaction with glucosamine molecules. Blue – human TLR4-MD-2 complex structure represented as ribbons; Red – interacting protein residues and glucosamine molecules with the latter shown as CPK, scaled to the number of molecules at each spot (0.9 for small spots, 1.7 for medium spots and 2 for large spots). The black ellipse highlights MD-2.

We then studied the possible binding modes between the LPS recognition system and structures of both the biologically inactive unmodified dendrimer and the biologically active partially glycosylated dendrimer. By subtracting the results for the unmodified dendrimer from those for the partially glycosylated dendrimer, it was possible to determine the contribution of the surface glucosamine molecules. The first studies involved the docking of the glucosamine molecules (as the moiety most likely to contribute to the biological activity of the partially glycosylated dendrimer) with the human TLR4-MD-2 complex (PDB entry: 3FXI) using Glue. As the binding site for glucosamine is not known, the whole TLR-MD-2 complex was used as a target by employing a set of overlapping boxes to increase the resolution of the docking solution. The results of this series of docking studies were overlapped and the solutions ranked according to the interaction energy observed (Figure 1).

The multiple binding sites of glucosamine molecules and their distribution on the surface of the protein complex meant that they were not suitable for the use of a “grow-out” strategy to determine the interactions between these molecules [23]. Nevertheless, it was notable that the three sites with a high number of poses and with high favourable interaction energies of the glucosamines with the TLR4-MD-2 complex were found to be along its beta-6 strand, the beta-7 strand, and the loop between amino acids Ile94 and Phe104. They line the opening of MD-2's hydrophobic pocket (Figure 1). These results led us to focus our docking studies with the whole dendrimer on MD-2 as the primary target.

MD-2 is a 160 amino acid glycoprotein with a MWt of 20 kDa [14]. It represents a class of MD-2–related lipid recognition (ML) proteins. It is folded into a single domain that consists of two ß sheets in the immunoglobulin fold. One sheet consists of three antiparallel ß strands, and the other sheet consists of six antiparallel strands. Between these sheets is a β cup topology lipid binding pocket with a volume of 1,710 Å^3^ and approximate dimensions of 15 Å by 8 Å by 10 Å. The ß6 and ß7 strands line the entrance to the hydrophobic pocket. Its shape suggests that it has evolved to accommodate large and structurally diverse ligands. Monomeric MD-2 binds to LPS and then forms a stable complex with TLR4 on the cell surface.

Initially, the interaction between these dendrimers and MD-2 (i.e., shape complementarity) was studied using the protein-protein interaction softwares Patchdock and Hex. They can carry out rigid docking only. In the absence of information about the dendrimer's bioactive conformation, we had to use different conformations of the dendrimers for these rigid docking studies. We had already shown that the partially glycosylated dendrimer was very flexible by molecular dynamics simulations of fully solvated dendrimers [7], [8]. Initial 3D structures for the simulation were generated using our “sequence to conformation” method [7], [8]. Twenty representative conformations were then selected from these molecular dynamics trajectories.

Both the unmodified dendrimer and the dendrimer with 8 surface glucosamines were docked as ligands using Patchdock and Hex. The unmodified dendrimer was used as a negative control because it did not alter the biological activity of the TLR4-MD-2-LPS cell surface receptor complex. The target used was the crystal structure of human MD-2 (PBD entry: 2e59). For each ligand, the 20 lowest energy solutions were saved (a total of 400 solutions per dendrimer) and a rebol script implemented in the Vega ZZ interface used to process the data. The results were analysed in terms of the total number of interactions between atoms in the dendrimer and the residues of MD-2 for both the biologically inactive unmodified dendrimer, and the biologically active partially glycosylated dendrimer. Although both dendrimers revealed similar interaction profiles, the partially glycosylated dendrimer was found to have better shape complementarity, and it formed more interactions with MD-2. These results showed that the surface conjugated glucosamines were important for the dendrimer's interaction with MD-2.

**Figure 2:**
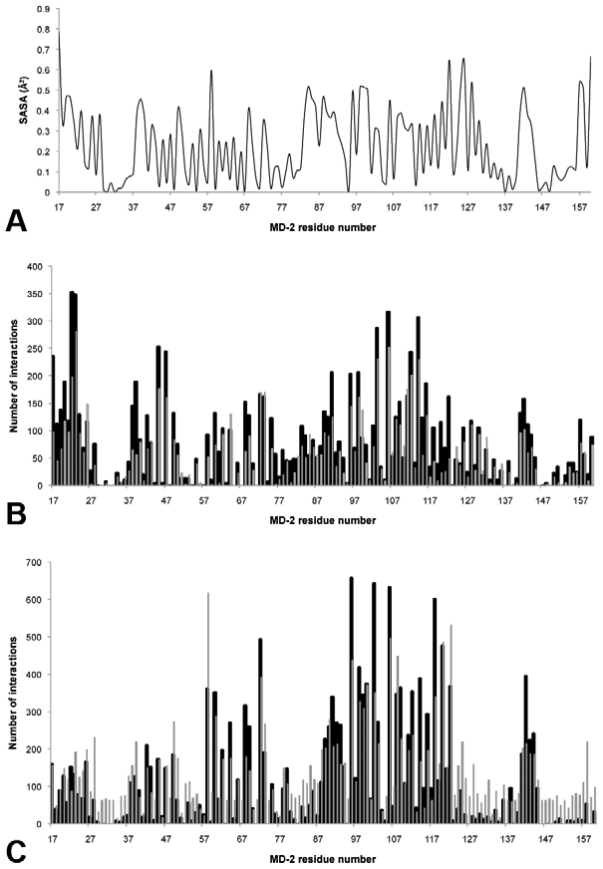
Interaction studies between the dendrimers and human MD-2. (A) Solvent accessible surface area (SASA) was determined for each human MD-2 residue; (B) Docking studies performed with Patchdock; (C) Docking studies performed with Hex. Black columns – dendrimer modified with 8 glucosamines. Grey columns – unmodified dendrimer.

To investigate if there was specificity in the interactions observed using Patchdock, the solvent accessible surface area (SASA) of each residue of MD-2 was determined (Figure 2A), and plotted alongside the number of interactions for the partially glycosylated dendrimer (Figure 2B). We found that the overall negative charge of the unmodified dendrimer's surface [7], [8] did not favour interactions with the negatively charged surface residues Glu92, Asp99 and Asp101 that line MD-2's hydrophobic pocket. This is the most likely explanation for the lack of biological activity of the unmodified dendrimer. Comparing the interaction profiles for unmodified dendrimers with those for partially glycosylated dendrimers also revealed that the most exposed residues were not always the ones with the highest number of interactions with MD-2. This indicated that there was selectivity in the interactions between the partially glycosylated dendrimer and MD-2. The same conformations of both dendrimers were then submitted for docking studies with the Hex shape and electrostatics protocol (Figure 2C). Although there was some similarity between the interaction profiles for Patchdock and Hex, a larger number of interactions were detected by Hex for both dendrimer types and the opening of MD-2's pocket. Comparing the results from the two software packages emphasised the importance of electrostatic interactions between the partially glycosylated dendrimer and MD-2.

**Figure 3:**
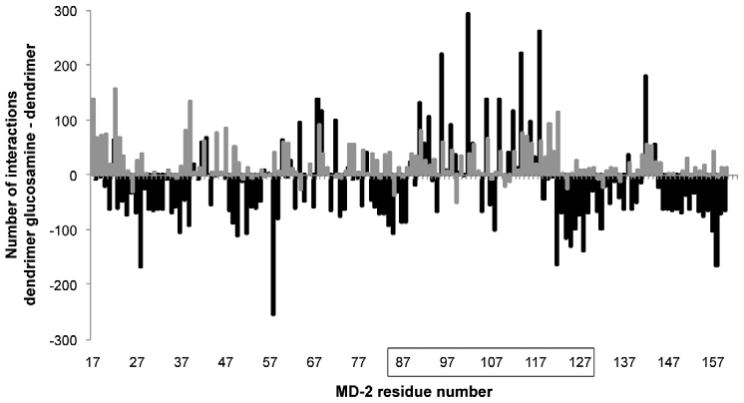
Difference between the number of interactions of human MD-2 with the unmodified dendrimer and the partially glycosylated dendrimer. Black columns – docking study performed with Hex; Grey columns – docking study performed with Patchdock. The Patchdock results showed a larger number of interactions with human MD-2 along its whole sequence. The Hex results showed a clear preference for interactions with residues lining the entrance to human MD-2's hydrophobic pocket (i.e., residues 84 to 127).

**Figure 4:**
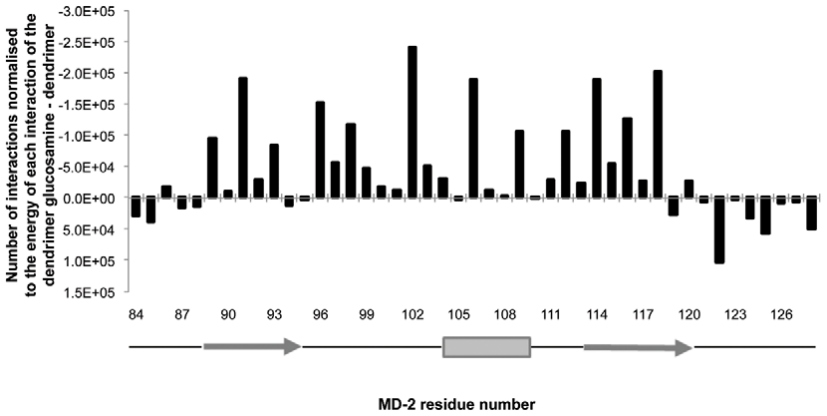
Number of interactions between human MD-2 and ligands normalised to the energy values for each interaction. The graph shows the difference between the number of interactions with human MD-2 of the unmodified dendrimer and the partially glycosylated dendrimer from the docking study performed with Hex after it was normalised for the energy value for each interaction. The graph also shows the residues that line the opening of human MD-2's hydrophobic pocket. The secondary structure of human MD-2 is shown with the solid grey arrows representing the ß6 (Pro88 to Ile94) and ß7 strands (Val113 to Ser120). The grey box represents the α-helix (Phe104 to Lys109). The lines represent the loops.

A subtraction of the profile for the partially glycosylated dendrimer from the unmodified dendrimer showed significant differences (Figure 3). Most of the interactions of the unmodified dendrimer were with residues located along the sides and back of MD-2's pocket. In contrast, the partially glycosylated dendrimer had multiple interactions with the entrance to the opening of MD-2's pocket. These results suggested that the biologically important interactions of the partially glycosylated dendrimer were occurring with residues that lined the entrance to MD-2's pocket. These encouraging results from Hex were then normalised with the energy value determined by multiplying the number of interactions by the corresponding interaction energy for each solution. When interaction energies were also taken into account, the differences between the unmodified dendrimer and the partially glycosylated dendrimer became even more marked for the residues lining the entrance to MD-2's hydrophobic pocket (Figure 4).

**Figure 5:**
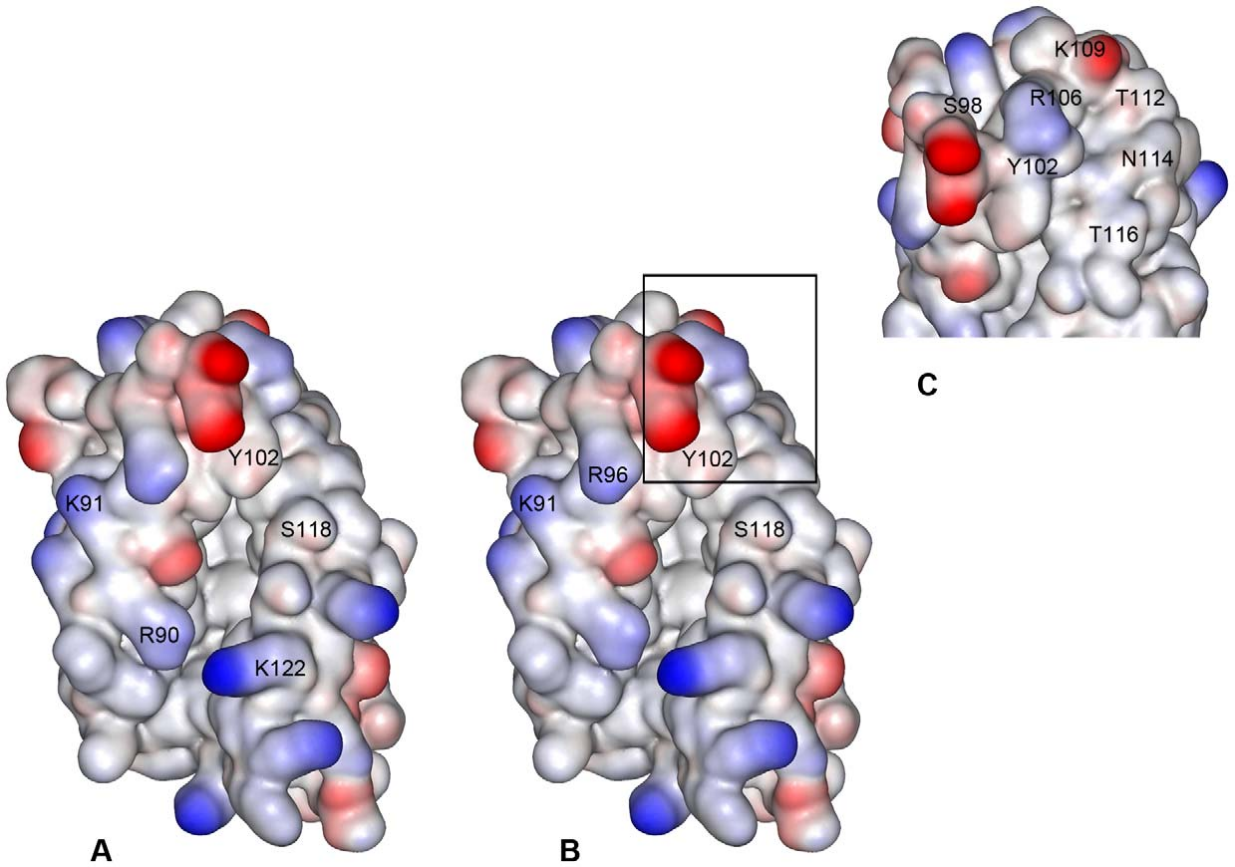
Crystal structure of human MD-2 with PDB entry 2E56 displayed as its electrostatic potential surface. (A – left hand side figure):- Residues Arg90, Lys91, Ser118 and Lys122 on human MD-2 form electrostatic interactions with lipid A. The interaction of Lys122 and Arg90 with the hydrophilic moiety of lipid A tethers LPS to human MD-2's cavity [15]. The 4′ phosphate on lipid A's first glucosamine binds to Ser118 and the 1′ phosphate on lipid A's second glucosamine binds to Lys122. In addition, Tyr102 is crucial for the subsequent hydrogen bond interaction of the human MD-2-LPS complex with human TLR4 [55]. (B – middle figure):- The residues Lys91, Arg96, Tyr102 and Ser118 form electrostatic interactions with the partially glycosylated dendrimer. (C – right hand side figure):- Human MD-2 is shown in its “top right” orientation. The residues Ser98, Tyr102, Arg106, Lys109, Thr112, Asn114 and Thr116 form electrostatic interactions with the partially glycosylated dendrimer. These residues, which border the entrance of human MD-2's hydrophobic pocket, are labelled in the figures.

The surface residues lining the entrance of human MD-2's pocket that have been shown to have a key role in the electrostatic binding of LPS are Arg90, Lys91, Ser118 and Lys122 (Figure 5A) [15], [17]. The biologically active partially glycosylated dendrimer showed the largest number and the strongest interactions with several of the residues lining the entrance to MD-2's pocket. Several of these residues are also important for the binding of LPS to MD-2. The residues with the highest normalized interaction values were Lys91, Tyr102, Arg106, Asn114 and Ser118. Several other residues, with lower interaction values, also contributed significantly to the co-operative binding of the partially glycosylated dendrimer to human MD-2; they were Arg96, Ser98, Lys109, Thr112 and Thr116 (Figures 5B & C).

**Figure 6:**
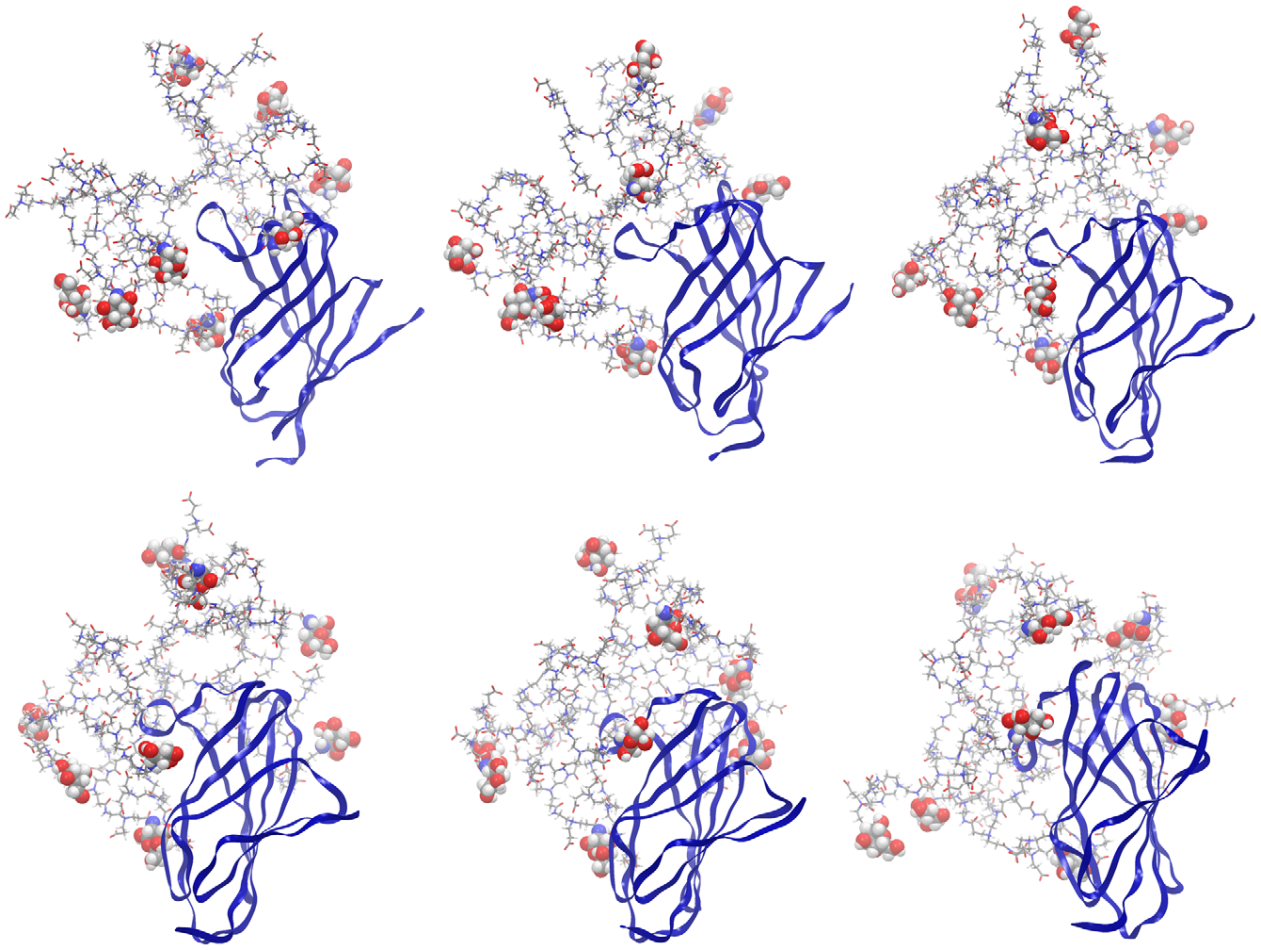
Snapshots of a molecular dynamics simulation of the partially glycosylated dendrimer with human MD-2 obtained with Hex. These figures show the molecule blocking the entrance to human MD-2's hydrophobic pocket. Top left and across the page to top right:- 0 ns; 0.8 ns and 1.6 ns. Bottom left and across the page to bottom right:- 2.4 ns; 3.2 ns and 4 ns. Human MD-2 is displayed as ribbons, the partially glycosylated dendrimer as tube, and the surface glucosamines as CPK.

**Figure 7:**
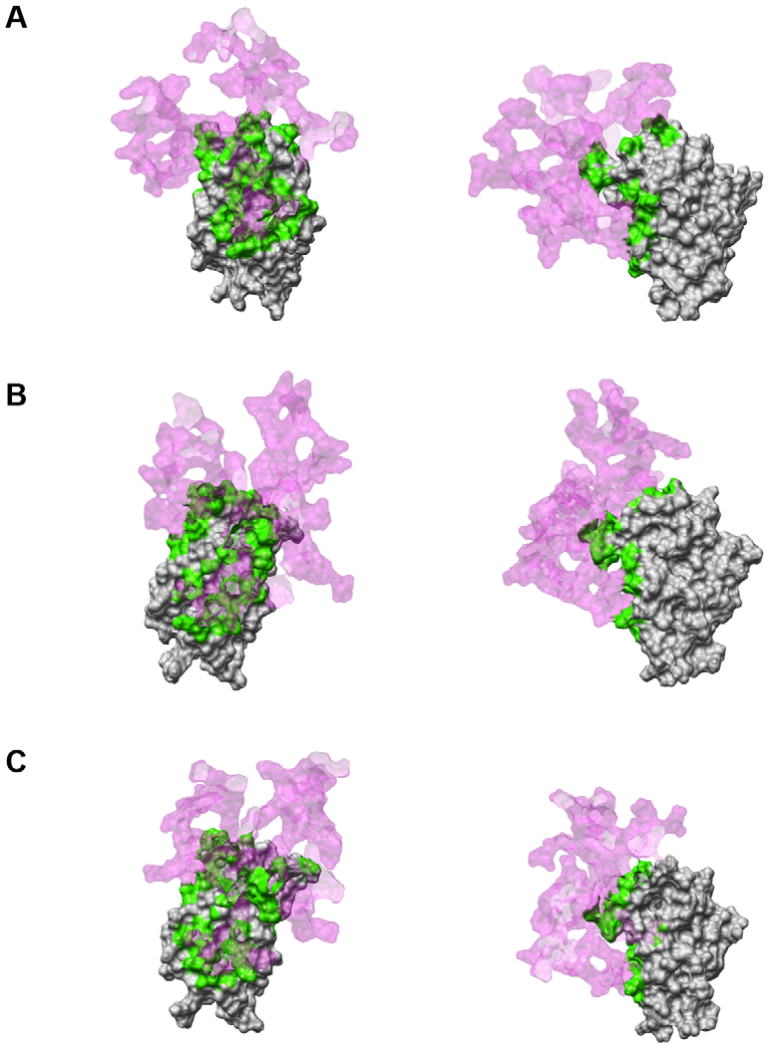
Surface contact area between human MD-2 and the partially glycosylated dendrimer. (A) 0 ns, (B) 1.6 ns, (C) 2.4 ns. Left – frontal view of human MD-2. Right – side view of human MD-2. The human MD-2 surface is shown in grey, the partially glycosylated dendrimer's surface is shown in pink with 80% transparency, and their contact surface area is shown in green.

To assess the dynamic behaviour of these interactions, and to determine whether using docking software designed for studying protein-protein interactions was affecting the results, a 4.8 ns molecular dynamics simulation of the partially glycosylated dendrimer complexed with MD-2 was performed in explicit solvent. The starting conformation used was one of the most stable docking solutions obtained with Hex. In this docking solution structure, the partially glycosylated dendrimer was located directly in front of MD-2's pocket with three of the glucosamine molecules in close proximity at 0 ns (Figure 6). As the simulation progressed, the partially glycosylated dendrimer moved away from MD-2 in order to rearrange its peripheral branches. This changed the number of glucosamine molecules facing MD-2's pocket. From 2.4 ns until the end of the simulation (i.e., 4.8 ns), the partially glycosylated dendrimer underwent further conformational changes such that 4 glucosamine molecules were consistently in close contact (i.e., 1.3 Å) with the entrance of MD-2's pocket (Figure 6). In addition, the entrance to this pocket was occluded by the partially glycosylated dendrimer during the entire 4 ns simulation. This is shown in Figure 7 by the semi-transparent pink colour which represents the partially glycosylated dendrimer's structure during its interaction with the entrance of MD-2's hydrophobic pocket.

**Figure 8:**
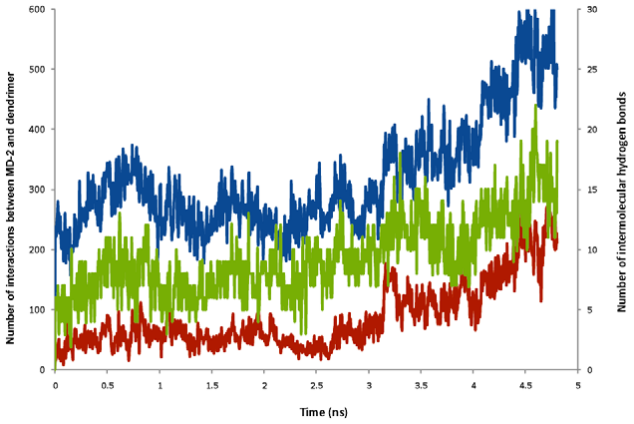
Interactions observed during a 4.8 ns molecular dynamics simulation of the partially glycosylated dendrimer – human MD-2 complex. Red – number of contacts of 1.3 Å between the dendrimer's surface glucosamine molecules and human MD-2. Blue – number of contacts of 1.3 Å between the partially glycosylated dendrimer (taking the molecule in its entirety) and human MD-2. Green – number of intermolecular hydrogen bond contacts between the partially glycosylated dendrimer (taking the molecule in its entirety) and human MD-2. Taken together, these results show that the affinity of the partially glycosylated dendrimer for human MD-2 involved both the dendrimer's surface glucosamine molecules and several of its peripheral carboxylic acid branches. Notably, these hydrogen bond interactions increased with time. This enabled the partially glycosylated dendrimer to block the entrance to human MD-2's hydrophobic pocket and thereby prevent access of the lipid chains of LPS.

**Figure 9:**
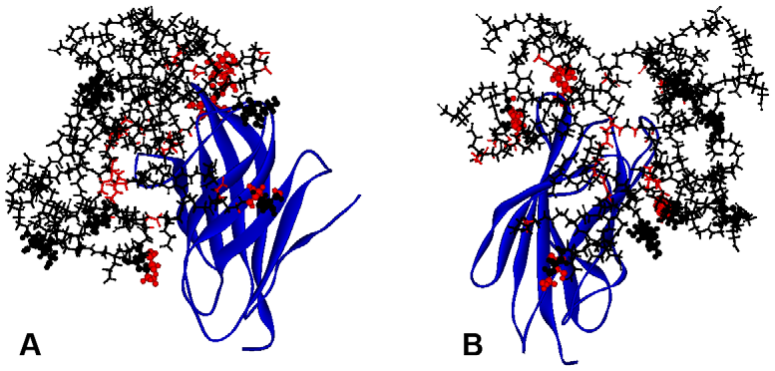
Interaction of the partially glycosylated dendrimer's surface with human MD-2's surface. Residues shown in red are <1.3 Å away from the surface of human MD-2. Glucosamine residues are shown in ball and stick configuration. (A) Side view of human MD-2's pocket; (B) frontal view of human MD-2's pocket.

**Figure 10:**
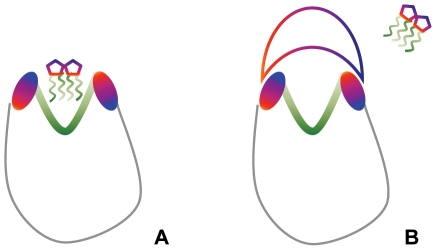
Schematic representation of the proposed mechanism of action of partially glycosylated dendrimers on human MD-2. Green – hydrophobic residues. Red and blue – hydrophilic regions.

The affinity of the partially glycosylated dendrimer for human MD-2 was demonstrated by the increased number of close contacts (i.e., 1.3 Å) between these two molecules (Figure 8) that involved both the glucosamine molecules and several of the dendrimer's peripheral carboxylic acid branches (Figure 9). These electrostatic interactions occluded the entrance to human MD-2's hydrophobic pocket and blocked access of the lipid chains of LPS. When taken with our previous observations that partially glycosylated dendrimers are both flexible and dynamic, this meant that conformational changes could induce shape complementarity [7], [8]. This enabled the dendrimer's surface glucosamine molecules to block the entrance of human MD-2's pocket. Additional co-operative electrostatic interactions with some of the dendrimer's free carboxylic acid branches follow. Collectively, they block the entry of the lipid chains of LPS into human MD-2's pocket, and also prevent TLR4-MD-2-LPS cell surface complex formation. This is shown schematically in Figure 10. The biologically important outcome is that the pro-inflammatory cytokine cascade is not initiated.

Lipid A is the primary immuno-stimulatory core of LPS. Although diverse across bacterial species, its di-glucosamine portion is universally conserved. Variants of LPS can be discriminated by the TLR4-MD-2 complex as being either endotoxic (i.e., agonist) or anti-endotoxic (i.e., antagonist) or partial antagonist/partial agonist. For example, lipid A (with six acyl chains) from *Escherichia coli* is a potent agonist of cytokine production in human and mouse macrophages [28]. The lipid chains form co-operative interactions with hydrophobic residues in MD-2's pocket. However, its precursor, lipid IVa, which has only four acyl chains, is an antagonist in human macrophages but an agonist in mouse macrophages. These lipid chain based differences have been used to define the central role of the di-glucosamine moiety with respect to the initial binding of LPS to MD-2, and the subsequent formation of the TLR4-MD-2-LPS complex [17]. Furthermore, the binding of all agonists to MD-2 induces a conformational change in MD-2, but the fact that such structural change does not occur when an antagonist binds to MD-2 has provided the basis for the structure-function based development of new molecules to modify this protein's biological activity. In this context, the key observation has been that underacylated lipid A functions as an antagonist by occupying MD-2 and not inducing the conformational change in this protein required to trigger TLR4 activation and oligomerisation [29]. The most advanced antagonist in development is eritoran (or e5564). It is a synthetic di-glucosamine and lipid based molecule that is derived from the lipid A structure of the nonpathogenic LPS of *Rhodobacter sphaeroides* [30]. It binds and blocks the entry of LPS into MD-2's pocket and thereby prevents formation of the TLR4-MD-2-LPS cell surface receptor complex. Unlike lipid A, eritoran's antagonist activity depends solely upon electrostatic interactions with Lys122 and Lys125 on the β-7 strand of MD-2.

These published observations about the lipid A based bioactive site of LPS make our results surprising because they suggested that the presence of highly hydrophobic lipidic chains was a prerequisite for any agonist or antagonist to bind effectively to MD-2 [31]. However, our results show, for the first time, that the presence of lipidic chains is not an absolute requirement for an MD-2 antagonist or partial antagonist. Our molecular docking studies also show that these partially glycosylated dendrimers bound to human MD-2 and interfered with the electrostatic binding of:- (i) the 4′phosphate on the di-glucosamine of LPS to Ser118 on MD-2; (ii) LPS to Lys91 on MD-2; (iii) the subsequent binding of TLR4 to Tyr102 on the MD-2-LPS complex. These three residues line the hydrophilic entrance of MD-2's hydrophobic pocket (Figure 5). Additional polyvalent interactions between several other human MD-2 surface residues [i.e., Arg96, Ser98, Arg106, Lys109, Thr112, Asn114 and Thr116 (Figures 5b & c)] and several of the partially glycosylated dendrimer's peripheral carboxylic acid branches (Figure 9) enables it to position itself so that it always occludes the entrance to MD-2's pocket (Figure 10). This blocks the entry of the lipid chains of LPS into human MD-2's pocket and explains the molecule's antagonist activity.

Our results also suggest that the interaction of the G3.5 partially glycosylated dendrimer with human MD-2 is specific. This conclusion is supported by the experimental biological data that only the TLR4 mediated pathway is affected by this molecule [6]. It should be noted that the dendrimer's surface glucosamines interact in a dynamic manner with several of the surface residues of MD-2. This promiscuity of MD-2 is an important evolutionary feature and reflects the need of MD-2 to be able to recognise all of the diverse LPS structures synthesised by individual species of Gram negative bacteria. MD-2's promiscuity extends to the binding of endogenous host derived ligands that act as alert signals for tissue injury, such as enzyme derived fragments of hyaluronan from the extracellular matrix [32].

Our previous modelling studies suggested that a dendritic architecture was important for this molecule's biological activity [7], [8]. In addition, our previous biological studies [6] were preceded by unpublished observations with smaller generations of partially glycosylated PAMAM dendrimers; these molecules did not have the desired biological activity. As the synthesis of higher generation dendrimers (e.g., G4.5 and higher) is both expensive and leads to considerable polydispersity of the molecule, their biological activity was not tested. However, our findings, based upon the detailed modelling of the G3.5 partially glycosylated dendrimer, suggest that larger generations of these molecules should be biologically active because they explore a similar conformational space to the G3.5 partially glycosylated dendrimer.

We have also been investigating the use of other polyanionic dendrimers with both different cores and branching units, but with the same carboxylated surface. Having successfully glycosylated different generations of these dendrimers, we have found that only some of them exhibit the biological activity required (paper in preparation). We are coming to the conclusion that the main difference between the biologically active partially glycosylated dendrimers and biologically inactive molecules lies in their flexibility which, in turn, leads to changes in both their peripheral conformation and their surface electrostatic properties.

Several years ago, we came to recognize the potential therapeutic importance for making polyvalent medicines because of our studies into the receptor-ligand interactions that mediate HIV-1 entry into cells [33], [34]. We, and others, then realized that some aspects of cell surface mediated immuno-regulation depended upon co-operative electrostatic interactions between carbohydrates and proteins [35], [36]. This has now been shown to be the case for the receptor-ligand interaction between TLR4 and LPS by the demonstration of an exponential increase in binding affinity [37]. Another example is the observation that four oligosaccharides are required for a functional immunological response to inhibit the LPS - DC SIGN cell surface interaction; N-acetyl-glucosamine-galactose-glucosamine and fucose-glucosamine-N-acetylglucosamine are required rather than N-acetyl-glucosamine or galactose on their own [38].

Our proof-of-concept studies have therefore demonstrated that it is possible to design a hyperbranched macromolecule with a pre-defined biological activity. The crucial advantage of our approach is that it avoids the expensive and complex chemistry associated with preparing oligosaccharides. This is because we can systematically modify the chemical functionality of the peripheral groups of dendrimers for a pre-defined biological activity using the simplified chemistry of monosaccharides to present aminosaccharides to cell surface receptors. As a result, medicinal grade product synthesis becomes a realistic goal for undertaking cost-effective clinical trials. Protein based medicines have already achieved this for macromolecules of >20 kDa by interacting with multiple cell surface receptors. Therefore, the use of dendrimeric structures of 2–5 kDa resolves two major obstacles that have impeded the therapeutic progression of synthetic macromolecules:- (i) excessive structural heterogeneity and poor control of molecular weight distribution; (ii) side-effects from *in vivo* activation of both complement and coagulation pathways [33], [34].

Our approach also enables, for the first time, the adoption of existing molecular modelling software (originally designed for studying protein-protein interactions) for the study of the interactions of synthetic macromolecules with biologically important proteins. Taken together with our previous modelling studies, which rationalised the effects of pegylation on the structure and biological activity of both proteins and peptides [39]–[43], we now believe that the principles and computational approaches that we have described could be applied more broadly to achieve proof-of-concept for other macromolecular structures.

We have also provided new insights into the interactions between a synthetic hydrophilic macromolecule and a protein usually targeted by hydrophobic molecules. These observations, coupled with an increasing number of recently published studies that underscore the important functional role of electrostatic interactions in TLR-ligand interactions, should facilitate the design of novel macromolecules that are both chemically well defined and biologically useful for the therapeutic manipulation of important TLR based immuno-pathological pathways [44]. This is because the binding orientations amongst all TLRs are similar even though the residual interactions with their ligands are specific. There is also an increasing body of evidence to suggest that a network of hydrogen bonds controls the precise positioning of the ligand in a TLR, and that it is the molecule's precise positioning that determines the specificity of TLR-ligand complex mediated signalling events [45], [46].

Established drugs are typically small molecules with a MWt of <500 Da. Biopharmaceuticals with a MWt of >20 kDa that are agonists of naturally occurring biological pathways are already on the horizon as important new medicines. Designing and delivering a novel generation of well defined chemically synthesised molecules with MWts of 2–5 kDa that mimic important biological properties is the new challenge for chemical-biologists. We propose that chemically optimised dendrimers with well defined chemical and biological properties (which we also propose could be called “synthetic baby bios” (SBBs)) can now be designed using the computational biology approaches described. These molecules could be useful for treating a spectrum of infectious, inflammatory and malignant diseases.

## Methods

### Validation of Patchdock and Hex softwares

Hex and Patchdock are two different software packages developed for the study of protein-protein interactions. Their algorithms and scoring functions are different. Patchdock reads each structure and transforms it into Conolly surface representations [47], [48] that include flat, concave and convex patches [49]. The complementarities between patches are assessed and then given a scoring function which takes into account both shape fitting and desolvation energy; i.e., energy required to break molecule-solvent interactions. The results are cluster based on the RMSD values of each transformation [49].

The crystal structure of the mouse TLR4-mouse MD-2 complex (PBD entry: 2z64) was used to evaluate whether the software could reproduce the structure of the complex. The mouse TLR4-mouse MD-2 complex was used because, at that time, there was no crystal structure for the human cell surface receptor complex.

For the negative controls, the target used was the same as that used for the positive control docking experiments; i.e., mouse TLR4 from the X-ray structure with PDB entry 2z64. However, the ligand was not mouse MD-2 from the same crystal structure but instead human MD-2 from the two different crystal structures available (i.e., PDB entries: 3FXI and 2e56). Docking for these negative control experiments was carried out using the same parameters as were used for the positive control experiments.

In the first experiment to dock TLR4 with MD-2, all of the oligosaccharides found in the original PDB entry file were removed. This resulted in a best fit that showed MD-2 interacting with TLR4 on the inside of the horseshoe (Supplementary Figure S1-A; brown image) rather than sitting on top of the TLR4 extracellular domain. When the change in docking target was changed to a TLR4 possessing all its oligosaccharides, this produced a good superimposition between the crystal structure and the docking result (Supplementary Figure S1-B).

A third experiment was performed keeping only the oligosaccharides inside the horseshoe. This also showed that the MD-2 from the crystal structure overlay the docked MD-2 (Supplementary Figure S1-C). This result indicated that the presence of sugar molecules inside the horseshoe was crucial for the correct positioning of MD-2 in the TLR4-MD-2 complex.

In the negative control experiments (Supplementary Figure S2), the lack of shape complementarity between the mouse and human regions critical for the TLR-4 and MD-2 interaction was used to confirm that a cross-species analysis (i.e., mouse TLR4 with human MD-2) did not reproduce the orientation of mouse TLR4 with mouse MD-2. It was also notable that human MD-2, taken from the crystal structure of the human TLR4-human MD-2 complex, gave a better solution that the use of human MD-2 complexed to lipid A from the crystal structure with PDB file entry 2z59. These negative control experiments confirmed that Patchdock was suitable for more detailed studies once the target molecules had been correctly defined.

The Hex software package was developed for protein and nucleic acid interaction studies. It also allows modelling of small ligand-protein interactions using rigid docking. Hex is a fast Fourier transform (FFT) docking correlations based programme that uses soft polar Fourier correlations to minimise the computational time required to explore the Cartesian space [50], [51]. The protein's molecular surfaces are represented by an internal and an external “skin” that are each represented by a Fourier series, and comprise radial and spherical harmonic basis functions [50]. The electrostatics contribution is optional, when present, and is only taken into account in the final search [52]; it has a small weighting in the final scoring function. The resulting structures are ordered from lowest to highest energy, and then clustered with a 3 Å threshold for the main chain C_α_ - C_α_ RMSD values [52].

The same crystal structure of the mouse TLR4-mouse MD-2 complex (PDB entry: 2z64) was used to evaluate whether the software could reproduce the structure of this complex. The influence of the “shape only” parameters versus “shape and electrostatics” parameters was also assessed. Since Patchdock revealed the importance of the presence of the oligosaccharides inside TLR4's horseshoe, these were considered in the docking process with Hex.

The docking results of the mouse TLR4-mouse MD-2 complex showed that the Hex shape only protocol software was inadequate for describing the system correctly (Supplementary Figures S3-A and S3-C). If a contribution from electrostatics (even with a smaller weighting) was taken into account, the docking reproduced the crystal structure albeit with a small deviation, but maintained the correct interaction site of TLR4 with MD-2 (Supplementary Figure S3-B).

These negative control experiments with Hex showed that the lack of shape complementarity between the human and mouse regions of MD-2 critical for TLR4-MD-2 complex formation (i.e., a cross-species analysis using mouse TLR4 and human MD-2) did not reproduce the orientation of mouse TLR4 in relation to mouse MD-2 (Supplementary Figure S4). Therefore, these modelling results were consistent with the biological observation that:- (i) pro-inflammatory cytokines are produced when HEK293 cells are transfected with human TLR4 and human MD2 and then stimulated with LPS; (ii) pro-inflammatory cytokine production does not occur when HEK293 cells are transfected with human TLR4 and mouse MD2 and then stimulated with LPS [53]. There is therefore a lack of shape complementarity between the human and mouse regions of MD-2 critical for the TLR4-MD-2 interaction. These observations led us to conclude that the Hex shape and electrostatics protocol was the most suitable for our further studies.

### Validation of GRID software

For a better understanding of the contribution of electrostatics in the interaction of the dendrimer and the glycosylated dendrimer with the LPS recognition system, GRID software was used. GRID is a calculation based procedure [54]. It enables the determination of the energetically favourable binding sites on a molecule whose structure is known. GRID results can be visualized with the Gview application. It allows visualization of molecular interaction fields, GRID energy contributions due to atoms of the target, and molecular structures with distances, torsion, and dihedral angles. Glue is a GRID docking programme that is capable of finding potential interaction sites between a molecule, set as “target”, and a small molecule set as “ligand”. It requires the input of both the target and the 3D structures of the ligand. Its scoring function takes into account steric repulsion energy, electrostatics contribution, a dry parameter (which accounts for hydrophobic energy), and an additional hydrogen bonding charge reinforcing parameter.

The system used for Grid validation was human MD-2-lipid IVa (PDB entry: 2z59). The GRID results for the structure of the human MD-2-lipid IVa complex were very similar to the crystal structure of lipid A with human MD-2's hydrophobic pocket, with respect to both position and conformation (Supplementary Figure S5).

In addition, the number of interactions between the residues of human MD-2 and the atoms of the partially glycosylated dendrimer (as summarised from the 400 solutions of the docking study performed with Hex) were plotted together with the number of interactions between the residues of human MD-2 and the single lipid A structure obtained from the crystal structure of its complex with MD-2 [PDB entry: 2z59] (Supplementary Figure S6). This highlighted:- (a) the hydrophobic nature of the binding of the acyl chains of lipid A to residues 27 to 37 of human MD-2; (b) the hydrophilic nature of the binding of the partially glycosylated dendrimer to the entrance of human MD-2's hydrophobic pocket {residues 84 to 127}; and (c) the competitive nature of the binding of these two molecules to the entrance of human MD-2's hydrophobic pocket.

The negative control experiments used sucrose and maltose as ligands. In biological experiments, we first confirmed that these two ligands did not compete with the binding site of LPS on human MD-2 (unpublished observations). They were then docked against the same target; i.e., human MD-2 from the PDB file entry 2e59. The results with maltose and sucrose confirmed that their binding sites were distant to the binding site of the lipid A of LPS to human MD-2 (Supplementary Figure S7). Based on these results, we considered GRID to be the most suitable software for our further studies.

### Crystal structures

The structures of the proteins used for the docking studies were obtained through the RCBS website (www.rcsb.org). The crystal structures of the mouse TLR4-mouse MD-2 complexed with eritoran (PDB entry: 2z64), and the human MD-2 complexed with lipid IVa (PBD entry: 2e59) were used for validation of the docking software. The complex of human TLR4-human MD-2 (PDB entry: 3FXI) was used as a target for the docking studies; the non-glycosylated MD-2 without ligand was extracted from the crystal structure (PDB entry: 3FXI) and loaded into Maestro. Hydrogen atoms were then added and a molecular dynamics simulation performed with Desmond. The fully solvated system was built using the SPC solvation model and the size of the box determined automatically by creating a 10 Å buffer around the system being simulated. The molecular dynamics simulation was performed for 200 ps at 300K and 1.03 bar. These included structure minimisation and relaxation steps. The final structure was used as a target in the docking studies performed with Patchdock and Hex.

### Docking studies with GRID22 software

Greater (Molecular Discovery Ltd) was used to generate the “.kout” files for all of the molecules studied. Glue (Molecular Discovery Ltd) was used for docking studies. For the docking process itself, all available probes were selected to generate molecular interactions fields (MIFs). A maximum of 100 binding sites were used with an energy cut-off of −100 kCal/mol. The maximum iterations value was set to 120. For ligand flexibility, 5 rotatable bonds were allowed and the electrostatic term was included for the calculation of the interaction energies. Glucosamine was docked with the human TLR4-MD-2 complex (PDB entry: 3FXI) using several overlapping target volumes defined as a box with a side of 30 Å.

### Docking studies with Patchdock

Patchdock was used with all default parameters, except that the maximum surface overlap had to be changed to the lower value of −2. The 20 lowest energy structures were saved as PDB files for further studies. The same protocol was used to dock mouse TLR4 with mouse MD-2 for validation, and to compare it with the crystal structure (PDB entry: 2z64). Once validated, the protocol was used to study the interactions of the G3.5 PAMAM dendrimer and the G3.5 partially glycosylated PAMAM dendrimer with:- (i) the human TLR4 - human MD-2 complex; (ii) human MD-2.

### Docking studies with Hex 5.0

The docking studies carried out with Hex involved the use of two different protocols. The first was a shape based protocol. The second included both shape and electrostatics based protocols. All other parameters were the same for both protocols and were set to their default values. The scan steps were set to 0.75 with 2 substeps. The order of the docking correlation was set to 25 for the steric scan, and 25 for the fine search. The grid size was set to 1 and 100 solutions. Of these, the representative structures of the first 20 clusters were saved as PDB files for further analysis. The studies of the unmodified dendrimer and the partially glycosylated dendrimer against human MD-2 and the human TLR4-human MD-2 complex were performed with the shape and electrostatics protocol. Twenty different conformations of each biologically inactive and active molecule were used as ligands.

### Data analysis of the docking solutions

For the visualisation of the outputs from the docking studies and to generate images, Discovery Studio Visualise v2.5 (Accelerys) was used. To process the 800 solutions resulting from the docking of the unmodified and modified molecules with human MD-2, a rebol script implemented in VegaZZ was used. This script reported every atom in the dendrimer that was at a distance of <3 Å from an atom in human MD-2. The resulting files were exported to Microsoft Excel and plotted as graphs. For energy scaling of the interactions, the number of interactions from each solution was multiplied by their energy value. The energy calculations were only performed for the Hex solutions.

### Molecular dynamics simulations

Initial conformations of the unmodified dendrimer and the partially glycosylated dendrimer were generated with X-PLOR using our “sequence to conformation” method [7], [8]. These structures and the complex of human MD-2 with the partially glycosylated dendrimer were obtained from a docking solution using Hex imported into Maestro, and then set up for molecular dynamics simulation with Desmond. The fully solvated systems were built using the SPC solvation model, and the size of the box determined automatically by creating a 10 Å buffer around the system being simulated. The molecular dynamics simulations were performed for 4.8 ns at 300K and 1.03 bar. These included structure minimisation and relaxation steps. Snapshot structures were recorded every 4.8 ps. The contact surface areas between the partially glycosylated dendrimer and human MD-2 were determined from selected frames of the 4.8 ns trajectory using the Chimera software package (www.cgl.ucsf.edu/chimera), having set the cut-off for van der Waal's interactions to 4 Å. The representative conformations of the unmodified dendrimer and the partially glycosylated dendrimer were obtained by trajectory analysis using Vega ZZ.

## Supporting Information

Figure S1 **Results of the interaction study of mouse TLR4 extracellular domain and mouse MD-2 with Patchdock.** Using as target – (A) mouse TLR4 in the PDB file entry 2z64 without any of the saccharides co-crystallised. Light blue – mouse TLR4; Dark blue – mouse MD-2 from the crystal structure; Brown – mouse MD-2 from the docked structure. (B) mouse TLR4 in the PDB file entry 2z64 with all of the saccharides co-crystallised. Purple – mouse TLR4; Dark blue – mouse MD-2 from the crystal structure; Brown – mouse MD-2 from the docked structure. (C) mouse TLR4 PDB file entry with only those saccharides inside the horseshoe that are close to where the interaction with mouse MD-2 is expected, and as co-crystallised and present in the PDB file entry 2z64. Light blue – mouse TLR4; Dark blue – mouse MD-2 from the crystal structure; Brown – mouse MD-2 from the docked structure.
(TIF)

Figure S2 **Negative control results obtained for the interaction study of the TLR4 extracellular domain and MD-2 with Patchdock.** (A) Docking results for mouse TLR4 with human MD-2 as complexed to human TLR4 (PDB entry: 3XFI). Light blue – mouse TLR4; Green – human MD-2 from the crystal structure of the human MD-2-human TLR4 complex. (B) Docking results for mouse TLR4 with human MD-2 as complexed with lipid A (PDB entry: 2E56). Light blue – mouse TLR4; Lime green – human MD-2 from the crystal structure of the human MD-2-lipid IVa complex.
(TIF)

Figure S3 **Results obtained for the interaction study of the mouse TLR4 extracellular domain and mouse MD-2 with Hex 5.0.** Using as target – (A) Crystal structure from the mouse TLR4-mouse MD-2 complex (PDB entry: 2Z64). Light blue – mouse TLR4; Dark blue – mouse MD-2 from the crystal structure. (B) Docking results obtained for the mouse TLR4-mouse MD-2 complex taking into account both shape and structure. Light blue – mouse TLR4; Dark blue – mouse MD-2 from the crystal structure; Brown – mouse MD-2 from the docked structure. (C) Docking results for the mouse TLR4-mouse MD-2 complex with a shape only protocol. Light green – mouse TLR4; Purple – mouse MD-2 from the crystal structure; Purple – mouse MD-2 from the docked structure. The saccharides in TLR4's horseshoe are hidden for clarity.
(TIF)

Figure S4 **Negative control results obtained for the interaction study of the TLR4 extracellular domain and MD-2 with Hex 5.0.** (A) Docking results for the mouse TLR4-human MD-2 complex. Light blue – mouse TLR4; Green – human MD-2 from the crystal structure of the human TLR4-human MD-2 complex. (B) Docking results for the mouse TLR4-human MD-2 complex. Light blue – mouse TLR4; Lime green – human MD-2 from the crystal structure of the human MD-2-lipid IVa complex. The saccharides in TLR4's horseshoe are hidden for clarity.
(TIF)

Figure S5 **GRID protocol validation.** (A) Crystal structure of the human MD-2-lipid IVa complex (PDB entry: 2e59). (B) Structure obtained with Glue for the same complex.
(TIF)

Figure S6 **Interaction studies of human MD-2 with lipid A and with the partially glycosylated dendrimer.** Number of interactions of <6 Å between human MD-2 residues and the atoms of:- (i) lipid A from the crystal structure of this complex (PDB entry: 2e59) as shown in red; (ii) the partially glycosylated dendrimer [as summarised from the 400 solutions of the docking study performed with Hex] as shown in black.
(TIF)

Figure S7 **Negative control experiments for GRID protocol validation.** (A) Docking results for maltose and human MD-2. (B) Docking results for sucrose and human MD-2. These experiments confirmed that the binding sites for maltose and sucrose on human MD-2 were distant to the binding site of the lipid A of LPS.
(TIF)
