# Supplementary figures and images for: Partially Glycosylated Dendrimers Block MD-2 and Prevent TLR4-MD-2-LPS Complex Mediated Cytokine Responses
Source: PLoS Comput Biol. 2011 Jun 30;7(6):e1002095. doi: 10.1371/journal.pcbi.1002095 (PMC3127813; doi:10.1371/journal.pcbi.1002095)

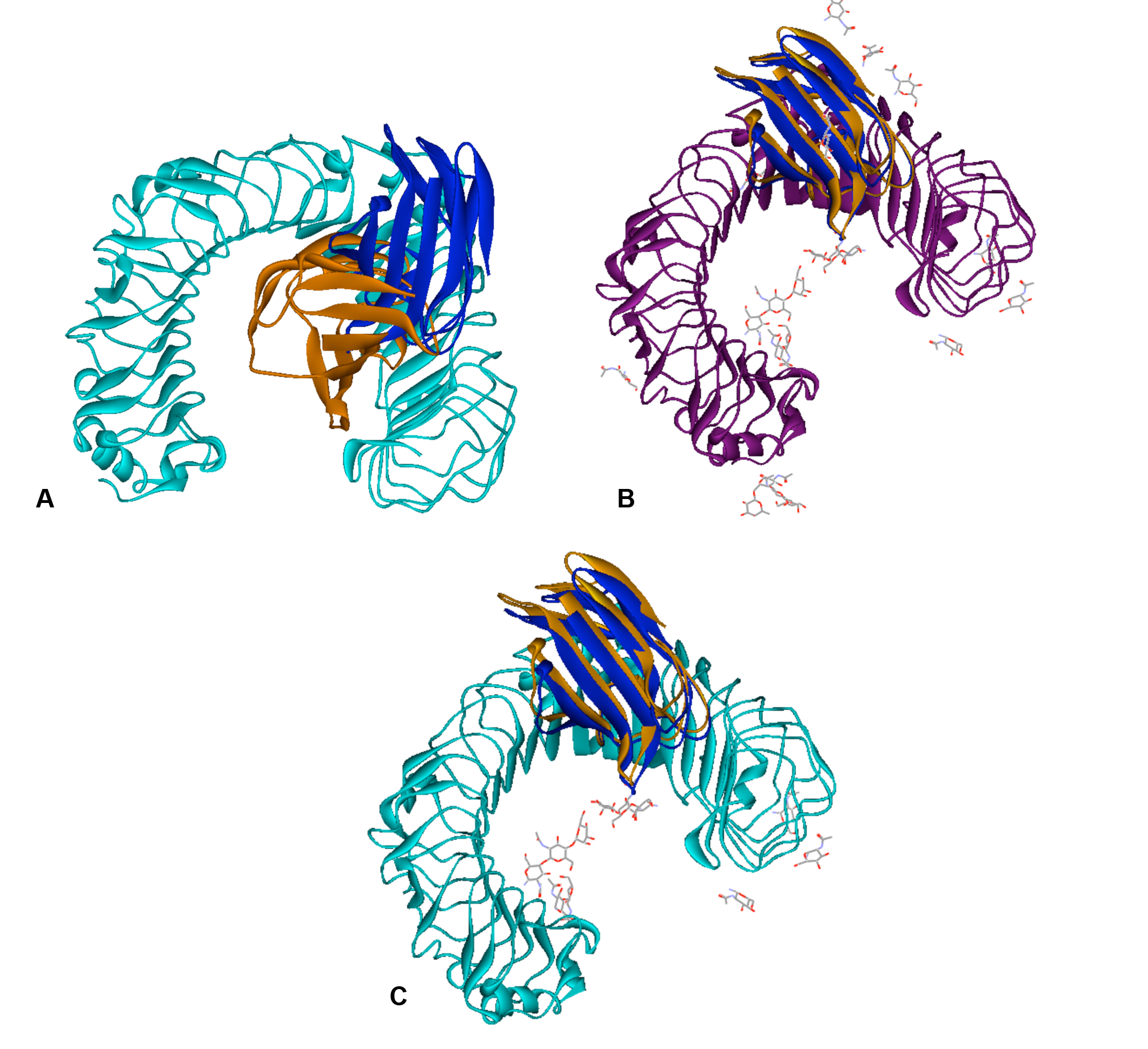

Supplement: Figure S1 — Results of the interaction study of mouse TLR4 extracellular domain and mouse MD-2 with Patchdock. Using as target – (A) mouse TLR4 in the PDB file entry 2z64 without any of the saccharides co-crystallised. Light blue – mouse TLR4; Dark blue – mouse MD-2 from the crystal structure; Brown – mouse MD-2 from the docked structure. (B) mouse TLR4 in the PDB file entry 2z64 with all of the saccharides co-crystallised. Purple – mouse TLR4; Dark blue – mouse MD-2 from the crystal structure; Brown – mouse MD-2 from the docked structure. (C) mouse TLR4 PDB file entry with only those saccharides inside the horseshoe that are close to where the interaction with mouse MD-2 is expected, and as co-crystallised and present in the PDB file entry 2z64. Light blue – mouse TLR4; Dark blue – mouse MD-2 from the crystal structure; Brown – mouse MD-2 from the docked structure. (TIF) [file pcbi.1002095.s001.tif]

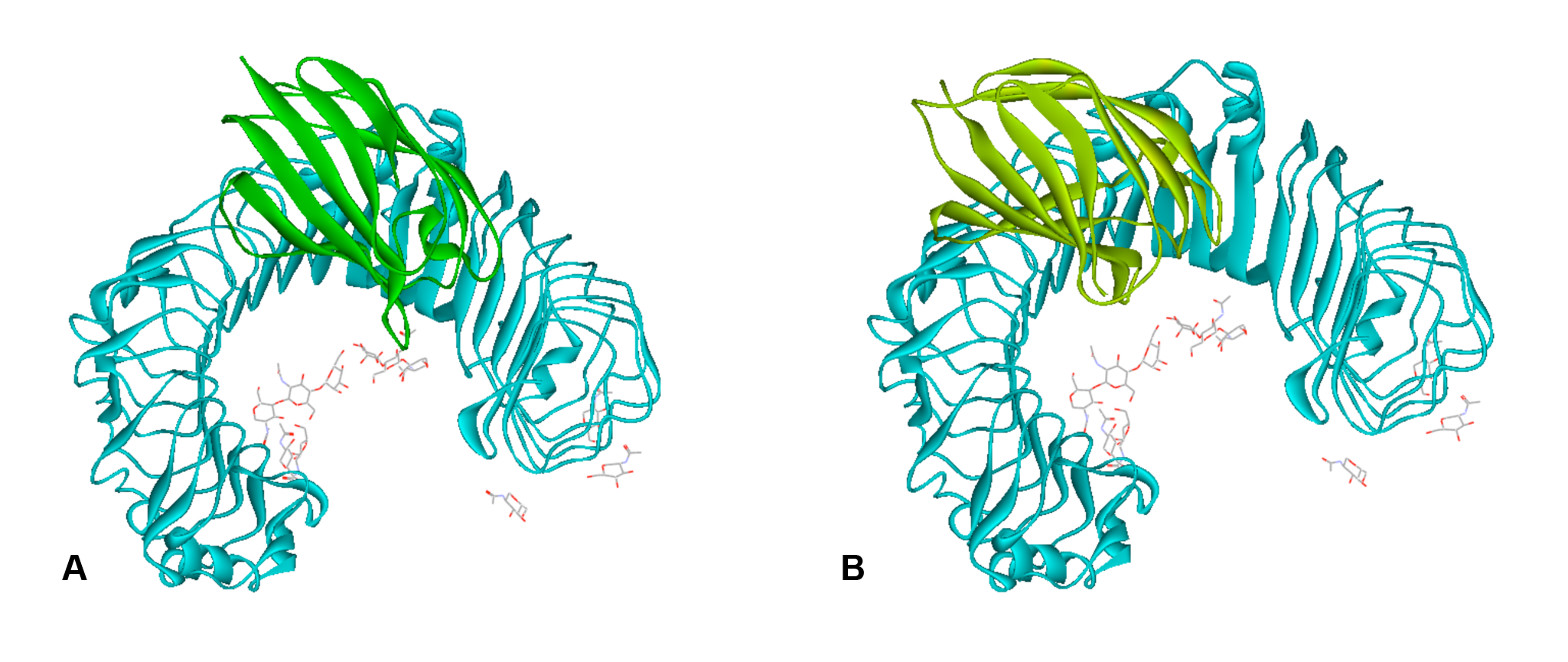

Supplement: Figure S2 — Negative control results obtained for the interaction study of the TLR4 extracellular domain and MD-2 with Patchdock. (A) Docking results for mouse TLR4 with human MD-2 as complexed to human TLR4 (PDB entry: 3XFI). Light blue – mouse TLR4; Green – human MD-2 from the crystal structure of the human MD-2-human TLR4 complex. (B) Docking results for mouse TLR4 with human MD-2 as complexed with lipid A (PDB entry: 2E56). Light blue – mouse TLR4; Lime green – human MD-2 from the crystal structure of the human MD-2-lipid IVa complex. (TIF) [file pcbi.1002095.s002.tif]

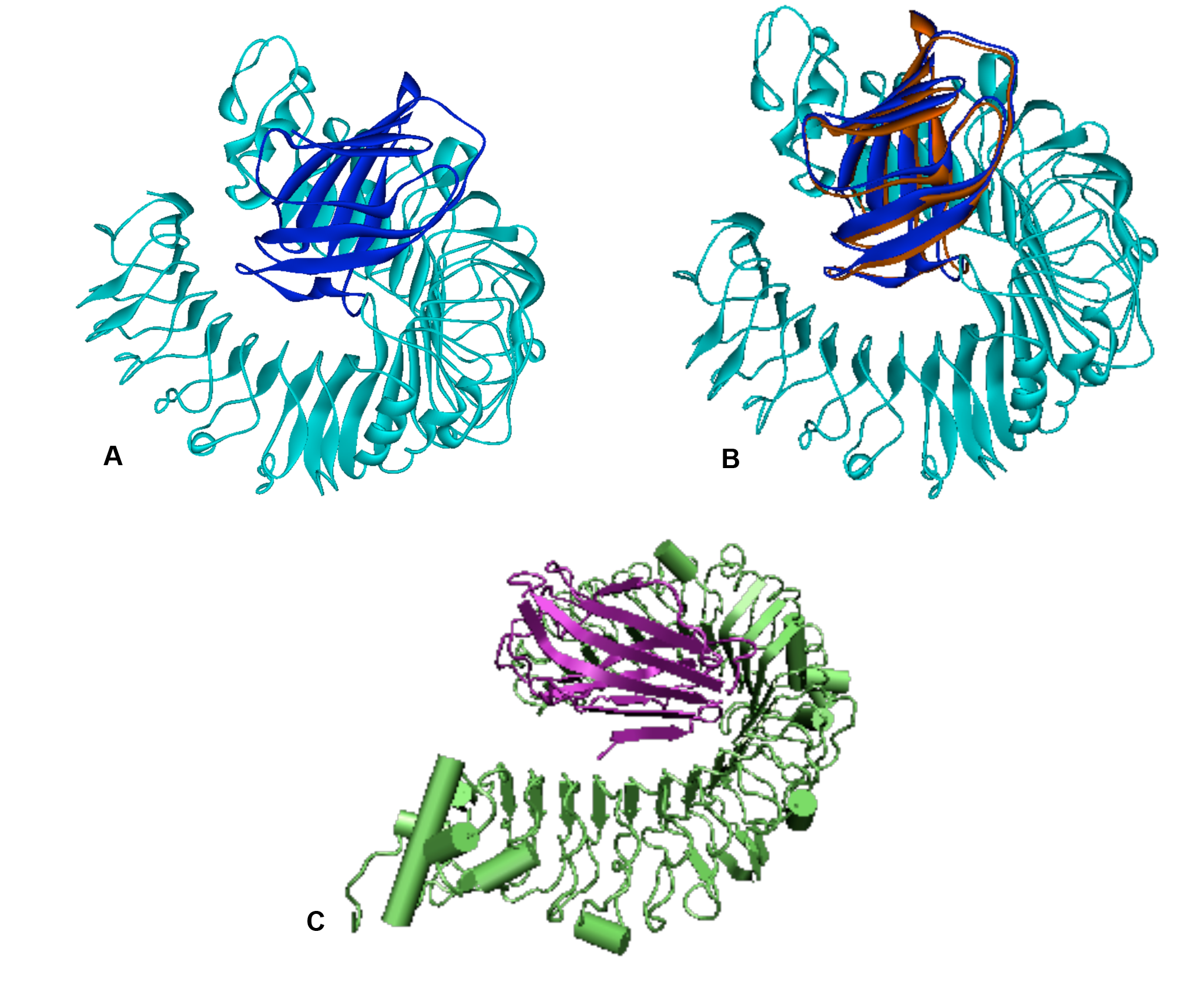

Supplement: Figure S3 — Results obtained for the interaction study of the mouse TLR4 extracellular domain and mouse MD-2 with Hex 5.0. Using as target – (A) Crystal structure from the mouse TLR4-mouse MD-2 complex (PDB entry: 2Z64). Light blue – mouse TLR4; Dark blue – mouse MD-2 from the crystal structure. (B) Docking results obtained for the mouse TLR4-mouse MD-2 complex taking into account both shape and structure. Light blue – mouse TLR4; Dark blue – mouse MD-2 from the crystal structure; Brown – mouse MD-2 from the docked structure. (C) Docking results for the mouse TLR4-mouse MD-2 complex with a shape only protocol. Light green – mouse TLR4; Purple – mouse MD-2 from the crystal structure; Purple – mouse MD-2 from the docked structure. The saccharides in TLR4's horseshoe are hidden for clarity. (TIF) [file pcbi.1002095.s003.tif]

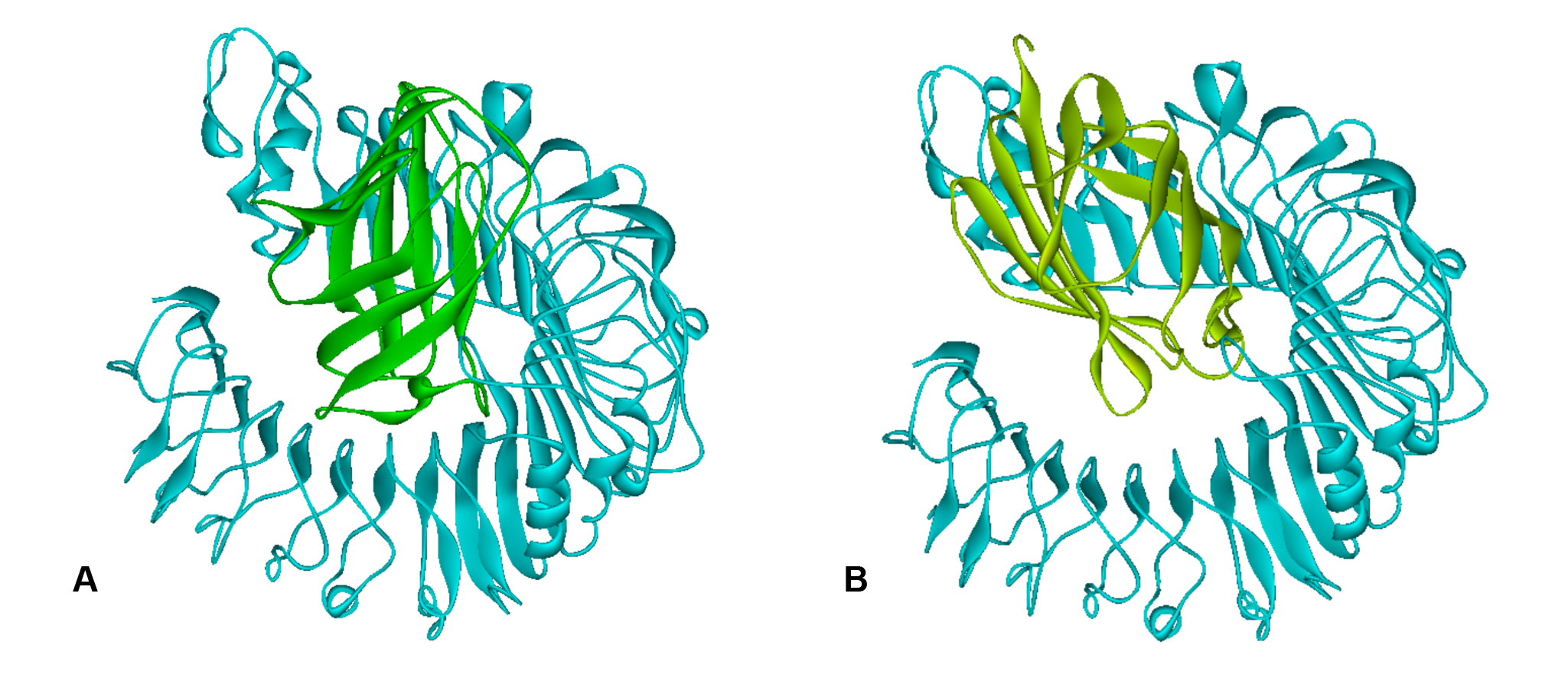

Supplement: Figure S4 — Negative control results obtained for the interaction study of the TLR4 extracellular domain and MD-2 with Hex 5.0. (A) Docking results for the mouse TLR4-human MD-2 complex. Light blue – mouse TLR4; Green – human MD-2 from the crystal structure of the human TLR4-human MD-2 complex. (B) Docking results for the mouse TLR4-human MD-2 complex. Light blue – mouse TLR4; Lime green – human MD-2 from the crystal structure of the human MD-2-lipid IVa complex. The saccharides in TLR4's horseshoe are hidden for clarity. (TIF) [file pcbi.1002095.s004.tif]

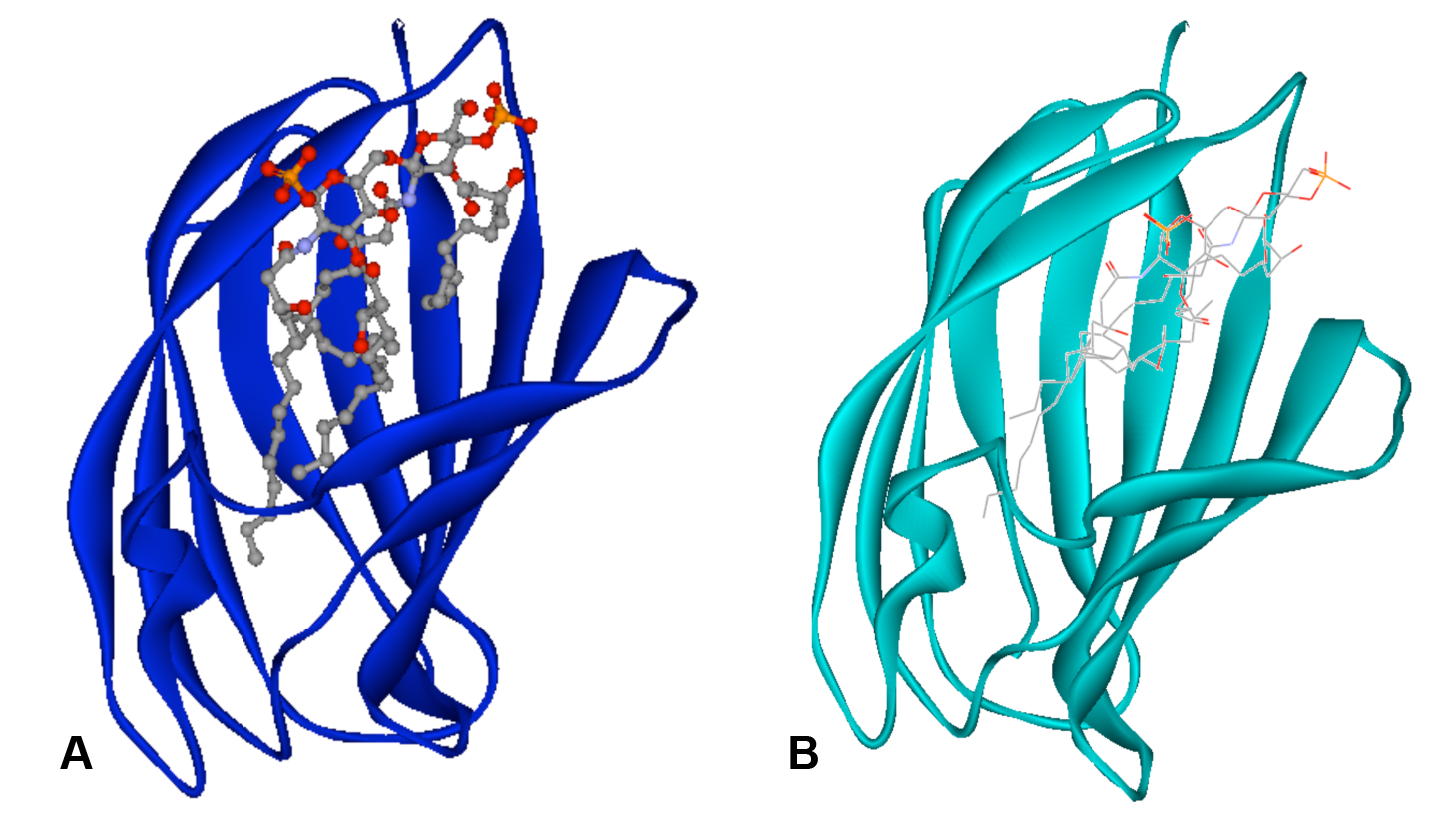

Supplement: Figure S5 — GRID protocol validation. (A) Crystal structure of the human MD-2-lipid IVa complex (PDB entry: 2e59). (B) Structure obtained with Glue for the same complex. (TIF) [file pcbi.1002095.s005.tif]

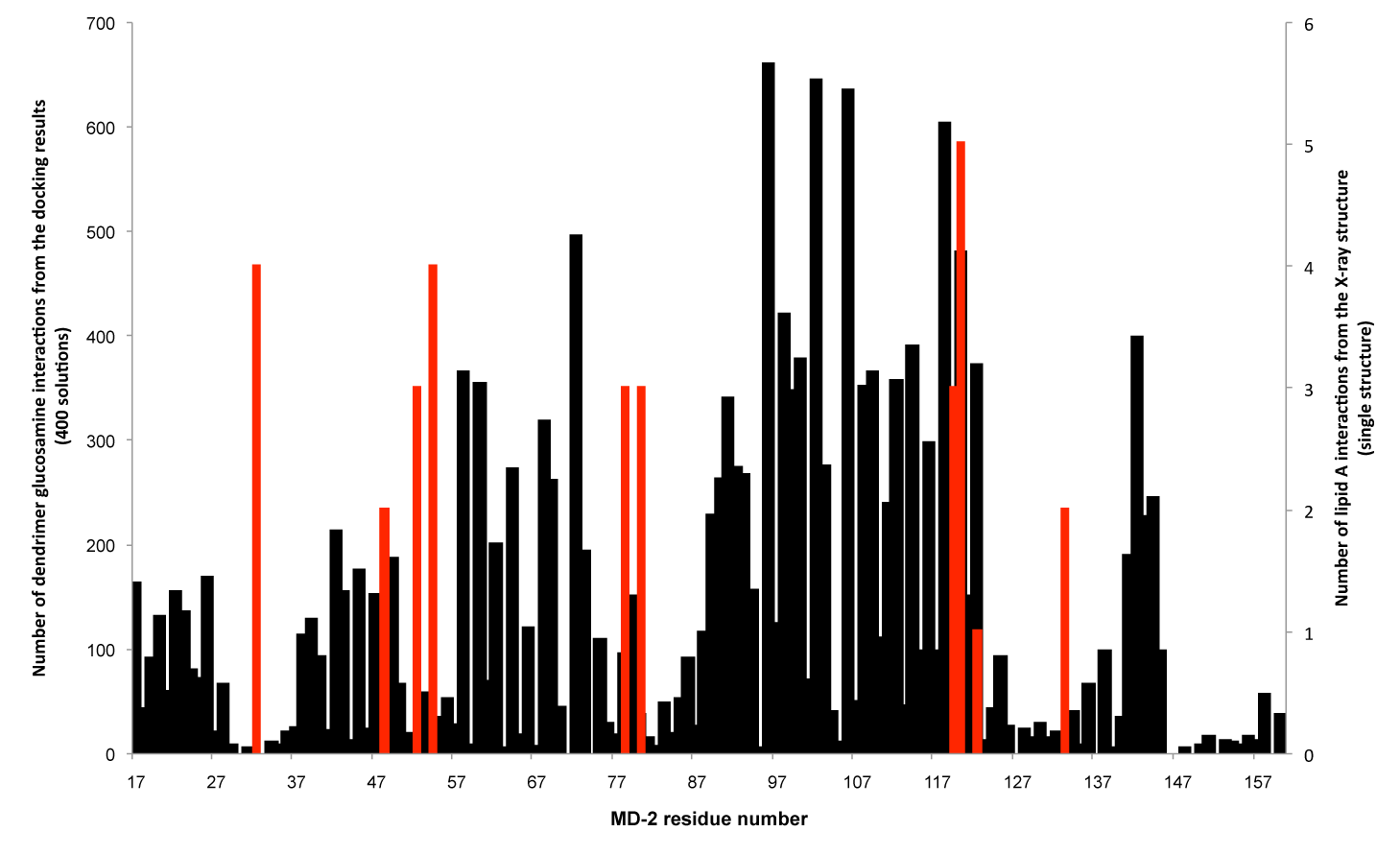

Supplement: Figure S6 — Interaction studies of human MD-2 with lipid A and with the partially glycosylated dendrimer. Number of interactions of <6 Å between human MD-2 residues and the atoms of:- (i) lipid A from the crystal structure of this complex (PDB entry: 2e59) as shown in red; (ii) the partially glycosylated dendrimer [as summarised from the 400 solutions of the docking study performed with Hex] as shown in black. (TIF) [file pcbi.1002095.s006.tif]

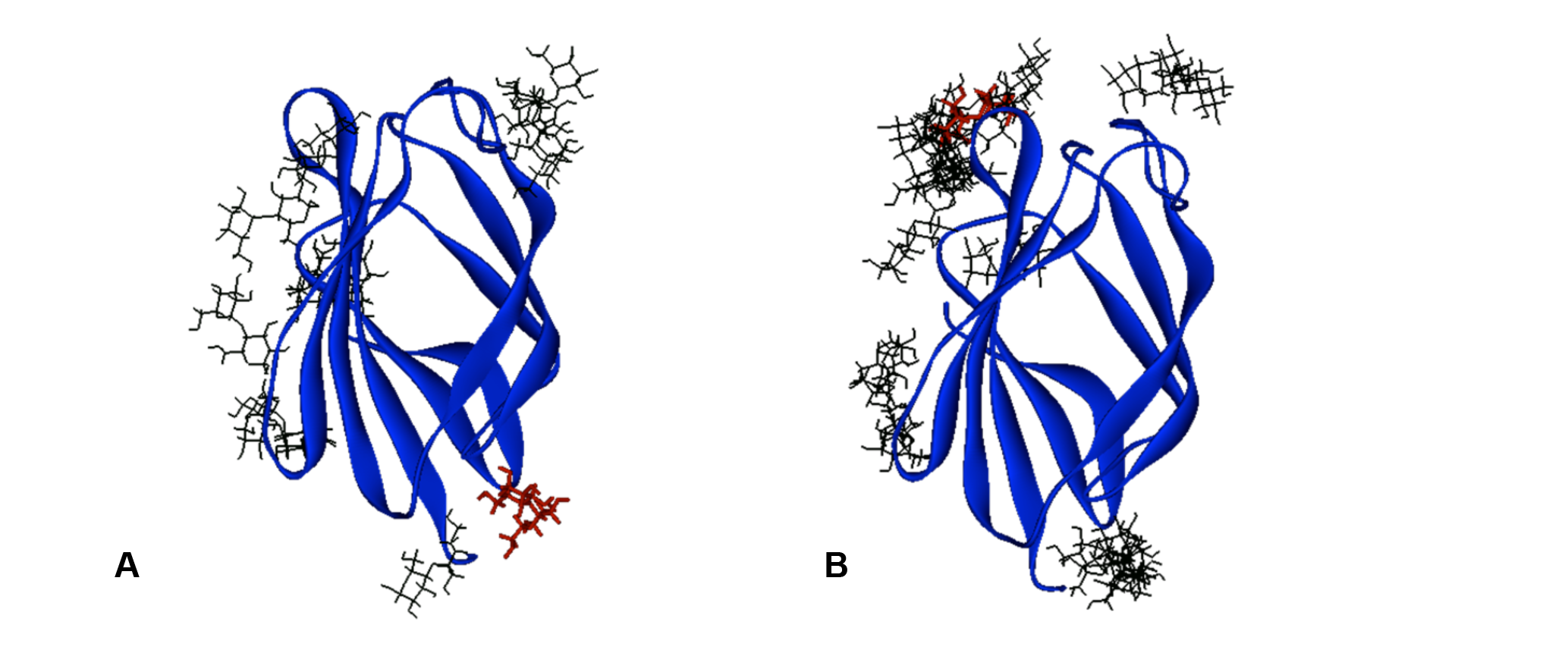

Supplement: Figure S7 — Negative control experiments for GRID protocol validation. (A) Docking results for maltose and human MD-2. (B) Docking results for sucrose and human MD-2. These experiments confirmed that the binding sites for maltose and sucrose on human MD-2 were distant to the binding site of the lipid A of LPS. (TIF) [file pcbi.1002095.s007.tif]
